# Simplified Acute Physiology Score 3 Performance in Austrian COVID-19 Patients Admitted to Intensive Care Units with and without Diabetes

**DOI:** 10.3390/v14040777

**Published:** 2022-04-08

**Authors:** Faisal Aziz, Alexander Christian Reisinger, Felix Aberer, Caren Sourij, Norbert Tripolt, Jolanta M. Siller-Matula, Dirk von-Lewinski, Philipp Eller, Susanne Kaser, Harald Sourij, on behalf of the COVID-19 in Diabetes in Austria Study Group

**Affiliations:** 1 Interdisciplinary Metabolic Medicine Trials Unit, Department of Endocrinology and Diabetology, Medical University of Graz, 8036 Graz, Austria; faisal.aziz@stud.medunigraz.at (F.A.); felix.aberer@medunigraz.at (F.A.); norbert.tripolt@medunigraz.at (N.T.); 2 Intensive Care Unit, Department of Internal Medicine, Medical University of Graz, 8036 Graz, Austria; alexander.reisinger@medunigraz.at (A.C.R.); philipp.eller@medunigraz.at (P.E.); 3 Division of Cardiology, Medical University of Graz, 8036 Graz, Austria; caren.sourij@medunigraz.at (C.S.); dirk.von-lewinski@medunigraz.at (D.v.-L.); 4 Division of Cardiology, Medical University of Vienna, AKH, 1090 Vienna, Austria; jolanta.siller-matula@meduniwien.at; 5 Center for Preclinical Research and Technology CEPT, Department of Experimental and Clinical Pharmacology, University of Warsaw, 02-672 Warsaw, Poland; 6 Department of Internal Medicine I, Medical University of Innsbruck, 6020 Innsbruck, Austria; susanne.kaser@i-med.ac.at

**Keywords:** SAPS 3, simplified acute physiology score, diabetes, intensive care unit, mortality, COVID-19, SARS-CoV-2

## Abstract

This study evaluated and compared the performance of simplified acute physiology score 3 (SAPS 3) for predicting in-hospital mortality in COVID-19 patients admitted to intensive care units (ICUs) with and without diabetes in Austria. The Austrian national public health institute (GÖG) data of COVID-19 patients admitted to ICUs (*n* = 5850) were analyzed. Three versions of SAPS 3 were used: standard equation, Central European equation, and Austrian equation customized for COVID-19 patients. The observed in-hospital mortality was 38.9%, 42.9%, and 37.3% in all, diabetes, and non-diabetes patients, respectively. The overall C-statistics was 0.69 with an insignificant (*p* = 0.193) difference between diabetes (0.70) and non-diabetes (0.68) patients. The Brier score was > 0.20 for all SAPS 3 equations in all cohorts. Calibration was unsatisfactory for both standard and Central European equations in all cohorts, whereas it was satisfactory for the Austrian equation in diabetes patients only. The SAPS 3 score demonstrated low discrimination and accuracy in Austrian COVID-19 patients, with an insignificant difference between diabetes and non-diabetes. All equations were miscalibrated particularly in non-diabetes patients, while the Austrian equation showed satisfactory calibration in diabetes patients only. Both uncalibrated and calibrated versions of SAPS 3 should be used with caution in COVID-19 patients.

## 1. Introduction

Coronavirus disease (COVID-19) has caused a devastating pandemic with a high hospitalization rate and mortality. As of 6 December 2021, more than 272 million cases of COVID-19 and more than 5 million deaths have been reported worldwide [1]. This health crisis has severely challenged the capacity of healthcare systems to treat hospitalized and critically ill COVID-19 patients [2]. In such a situation, prognostic scores may offer a cost-effective and practical strategy to prioritize and allocate health resources, guide patient management, and evaluate the effectiveness of therapeutic interventions in intensive care units (ICUs) [3].

Numerous scoring systems are routinely utilized in ICUs to evaluate the quality of care and predict the prognosis of patients [3]. However, these scores need to be updated regularly to adjust for diagnostic and therapeutic advances in the ICU practice and changes in disease patterns [3]. The simplified acute physiology score version 3 (SAPS 3) is one of the most widely used scores in the ICU and has been extensively validated in Europe and other regions. The SAPS 3 incorporates various ICU- and patient-related factors for assessing disease severity and predicting in-hospital mortality [4].

Despite the obvious benefits of prognostication scores, validation is required in COVID-19 patients before their application in this population. In this regard, a few studies have evaluated the performance of various scores including SAPS 3 in COVID-19 patients albeit with contradictory findings. The SAPS 3 has overestimated the mortality in high-risk Brazilian patients suffering from COVID-19, whereas it has significantly underpredicted the mortality in Austrian COVID-19 patients. In addition, calibration of SAPS 3 was inadequate in both Brazilian and Austrian COVID-19 patients [5,6]. These conflicting results warrant more validation studies of SAPS 3 in COVID-19 cohorts. In addition, as people with diabetes are regarded as a high-risk group for COVID-19 morbidity and mortality, no previous study has compared the performance of SAPS 3 in COVID-19 patients with and without diabetes. In this study, we evaluated the performance of SAPS 3 for predicting in-hospital mortality in a countrywide cohort of COVID-19 patients admitted to ICUs in Austria. In addition, we compared its predictive performance between patients with and without diabetes.

## 2. Methods

### 2.1. Study Design and Data Source

The “Transparent Reporting of a multivariable prediction model for Individual Prognosis Or Diagnosis (TRIPOD)” checklist was used for reporting this study [7]. This study retrospectively analyzed the cohort of patients with and without diabetes mellitus admitted to ICUs following primary or secondary diagnosis of SARS-CoV-2 infection from March 2020 to March 2021 in Austria. These data are collected and maintained by the “Data platform COVID-19” commissioned by the Austrian National Public Health Institute (Gesundheit Österreich GmbH, Vienna, Austria). This platform gathers nationally representative countrywide epidemiological and clinical data of COVID-19 patients to provide updated evidence on SARS-CoV-2 infection in Austria. The details of this data platform can be accessed at: https://datenplattform-covid.goeg.at/english (accessed on 3 May 2021).

### 2.2. Data Extraction

**Figure 1:**
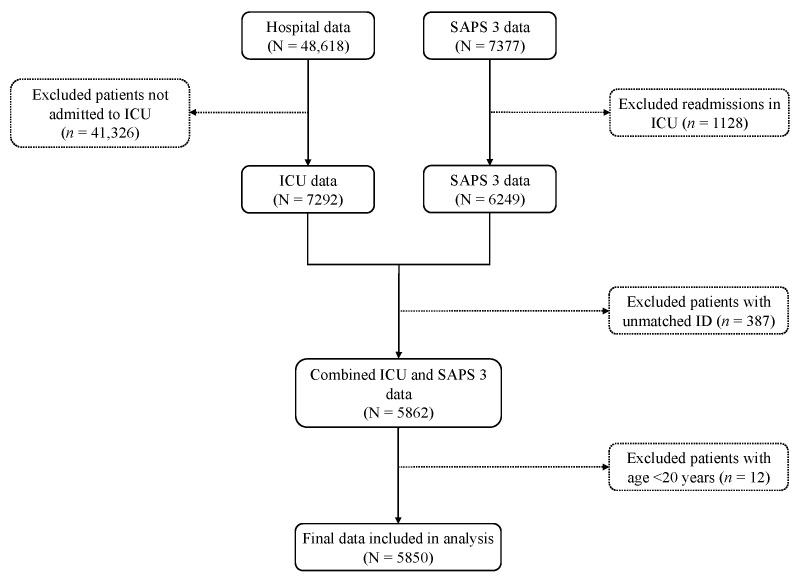
Flow diagram of data extraction. ICU, intensive care unit; SAPS 3, simplified acute physiology score 3.

For this study, two anonymized datasets were received from the Austrian data platform: (1) hospital data that comprised variables on demographic characteristics, comorbidities, ICU stay, and in-hospital mortality and (2) SAPS 3 data that comprised variables for calculating SAPS 3 score and the number of readmissions in the ICU. Data of patients admitted to ICU were extracted from the hospital data and then matched and merged with the SAPS 3 data after removing readmissions. Afterwards, patients aged less than 20 years were removed from the merged data, as only adults were considered in the study. A total of 5850 patients were included in the final analysis (Figure 1).

### 2.3. Study Variables

The outcome variable was in-hospital mortality, which was defined as death occurring in the hospital following hospitalization for primary or secondary SARS-CoV-2 infection or discharged alive from the hospital. Diabetes was recorded in the database as a comorbidity (insulin and non-insulin dependent diabetes) for the SAPS 3 and as per International Classification of Disease (ICD) version 10 codes (E10, E11, E12, E13, E14).

The SAPS 3 score consists of 20 variables that were recorded at the time of ICU admission. These variables were classified as patient characteristics, reasons for ICU admission, and acute physiological disruptions. Patient characteristics included age deciles (20–90+ years), gender, previous health status, comorbidities, intra-hospital location before ICU, length of stay in the hospital before ICU admission, and major therapeutic interventions before ICU admission. Reasons for ICU admission included health conditions, status and site of surgery, and the presence of infection at ICU admission. Acute physiological disruptions were measured in terms of vital signs, neurological status, serum creatinine, leukocytes, platelets, blood pH, partial pressure of oxygen (PaO_2_), and a fraction of inspired oxygen (FiO_2_). The detailed information regarding the calculation of the SAPS 3 score is published elsewhere [4].

### 2.4. Statistical Analysis

#### 2.4.1. Summary Statistics

Data were received in Microsoft Excel and analyzed in R version 1.4.1 and Stata version 17.0 (Stata Corp, Houston, TX, USA). Missing values of SAPS 3 variables were replaced with either reference or normal categories as recommended in the SAPS 3 publication. Continuous variables were reported as mean ± standard deviation (SD) or median and interquartile range (IQR) if not normally distributed. Categorical variables were reported as frequencies with corresponding percentages (%).

#### 2.4.2. Calculation of SAPS 3 Score and Predicted in-Hospital Mortality

The SAPS 3 score was calculated only for the first episode of ICU admission using the variables and algorithm recommended in the original publication [4]. The predicted in-hospital mortality was estimated from the SAPS 3 score using three logit regression equations: (1) standard equation (Logit = −32.6659 + ln [SAPS 3 score + 20.5958] × 7.3068); (2) Central European equation (Logit = −36.0877 + ln [SAPS 3 score + 22.2655] × 7.9867); and (3) recently published Austrian equation recalibrated for COVID-19 patients (Logit = −14.451 + ln [SAPS 3 score + −12.092] × 3.666) [4,6]. In addition, the standardized mortality ratio (SMR) was estimated by dividing the observed mortality rate with the predicted mortality rate with corresponding 95% confidence intervals (CI) to test the uniformity of fit. The value of SMR < 1 indicates overestimation, while >1 indicates an underestimation of the outcome.

#### 2.4.3. Assessment of Predictive Performance of SAPS 3

The predictive performance of each SAPS 3 equation was assessed in terms of discrimination, calibration, and accuracy. Discrimination was assessed by estimating the area under the receiver operating characteristics curve (AUC) or C-statistic with corresponding 95% CI. The AUC was compared between patients with and without diabetes using the DeLong test, and a *p*-value of <0.05 was chosen to determine statistical significance. The Youden index was estimated to select the optimal cut-off value of SAPS 3 score for the overall, diabetes, and non-diabetes cohorts. The identified cut-off values were then used to calculate sensitivity, specificity, and predictive values of SAPS 3 score.

Calibration was assessed by comparing the predicted probability against the observed probability using the Hosmer–Lemeshow (H-L) goodness-of-fit test and the calibration plot. In the H-L test, the *p*-value > 0.05 indicates a good fit. In the calibration plot, the calibration slope close to 1 indicates good calibration, the calibration intercept (calibration in the large (CITL)) close to 0 indicates good calibration, and the alignment of calibration lowess curve with the reference line indicates good calibration. Accuracy of the SAPS 3 in predicting in-hospital mortality was assessed using the Brier score. The Brier score ranges from 0 to 0.25, with 0 indicating perfect accuracy and 0.25 indicating non-informative accuracy.

### 2.5. Ethical Considerations

This study was approved by the Ethics Committee of the Medical University of Graz, Graz, Austria (ethics number 32-355 ex 19/20). This study followed the guidelines of good clinical practice and the Declaration of Helsinki 1964. No consent forms were obtained from the study participants, as it was a retrospective analysis of pseudonymized data.

## 3. Results

### 3.1. Characteristics of Patients

Table 1 shows the distribution of characteristics, SAPS 3 variables, and SAPS 3 score in COVID-19 patients admitted to ICU in all, diabetes, and non-diabetes patients. Of the 5850 patients admitted to ICU, 1667 (28.50%) had diabetes. Most patients were males (66.07%) and aged above 60 years. The mean ± SD SAPS 3 score was 57.39 ± 13.18 in the overall cohort and was significantly higher in patients with diabetes than those without diabetes (58.78 ± 12.92 vs. 56.84 ± 13.23, *p* < 0.001).

In reasons for admission, the category “all others” in each system include reasons that are either not related to that particular system or those not falling in specified categories within that system.

Pearson’s chi-square or Fisher’s exact test were applied to compare qualitative variables with diabetes status. Two sample *t*-tests or Wilcoxon rank sum tests were applied to compare quantitative variables with diabetes status.

### 3.2. Observed In-Hospital Mortality and Its Comparison with Variables

Table 2 shows that the overall observed in-hospital mortality was 38.91%, and it was significantly higher in patients with diabetes (42.95% vs. 37.29%, *p* < 0.001) compared to those without diabetes. Patients who died in the hospital had significantly higher mean ± SD SAPS 3 scores compared to those who were alive in all (62.57 ± 12.86 vs. 54.10 ± 12.29, *p* < 0.001), diabetes (63.96 ± 13.15 vs. 54.87 ± 11.28, *p* < 0.001), and non-diabetes patients each (61.92 ± 12.68 vs. 53.82 ± 12.62, *p* < 0.001).

In reasons for admission, the category “all others: in each system include reasons that are either not related to that particular system or those not falling in specified categories within that system.

Pearson’s chi-square or Fisher’s exact test were applied to compare qualitative variables with in-hospital mortality status. Two sample *t*-tests or Wilcoxon rank sum tests were applied to compare quantitative variables with in-hospital mortality status.

### 3.3. Predicted In-Hospital Mortality and Standardized Mortality Ratio

Table 3 shows that the mean predicted mortality estimated by the SAPS 3 standard equation in all patients was 32.47 ± 21.69, Central European equation was 28.05 ± 21.43, and Austrian equation was 37.86 ± 20.56. The predicted mortality was significantly higher in patients with diabetes compared to those without diabetes for standard (34.56 ± 21.62 vs. 31.63 ± 21.66, *p* < 0.001), Central European (30.02 ± 21.56 vs. 27.28 ± 21.33, *p* < 0.001), and Austrian equations each (40.03 ± 20.15 vs. 37.00 ± 20.66, *p* < 0.001). The SMR value > 1 with their corresponding CIs for both standard and Central European equations indicated that these equations significantly underestimated the in-hospital mortality in all three patient populations i.e., all, diabetes, and non-diabetes patients. The Austrian equation concorded well with the observed mortality in the overall and non-diabetes cohorts, whereas it slightly underpredicted the mortality in patients with diabetes.

### 3.4. Discrimination and Accuracy of SAPS 3

**Figure 2:**
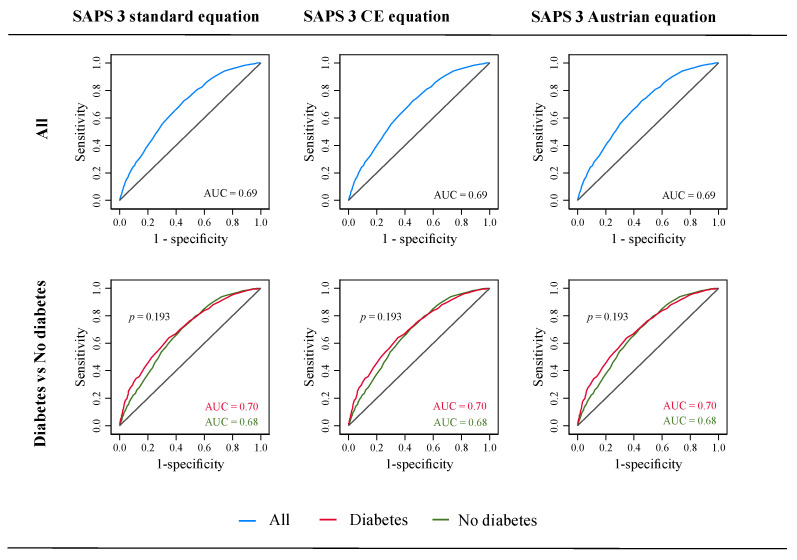
Receiver operating characteristics (ROC) curves for SAPS 3 standard, SAPS 3 Central Europe, and SAPS 3 Austrian equations in all, diabetes, and non-diabetes patients with COVID-19. AUC, area under the curve; CE, Central European; SAPS 3, simplified acute physiology score 3; *p*, *p*-value for DeLong test.

The optimal cut-off SAPS 3 score was 55, 55, and 58 for the overall, non-diabetes, and diabetes cohorts, respectively. Based on these cut-off scores, sensitivity was 72.4%, 70.6%, and 66.8%; specificity was 54.5%, 56.0%, and 60.6%; positive predictive value was 50.3%, 48.8%, and 56.0%; and negative predictive value was 75.6%, 76.2%, and 70.8% for the overall, non-diabetes, and diabetes cohorts, respectively. The SAPS 3 showed unsatisfactory discrimination for all three equations (AUC = 0.69) with an insignificantly (*p* = 0.193) higher discrimination in patients with diabetes (AUC = 0.70) compared to those without diabetes (AUC = 0.68) for each equation (Table 3 and Figure 2). The Brier score was > 0.20 for all three equations in three patient cohorts, which indicated its poor accuracy in COVID-19 patients (Table 3).

### 3.5. Calibration of SAPS 3

**Figure 3:**
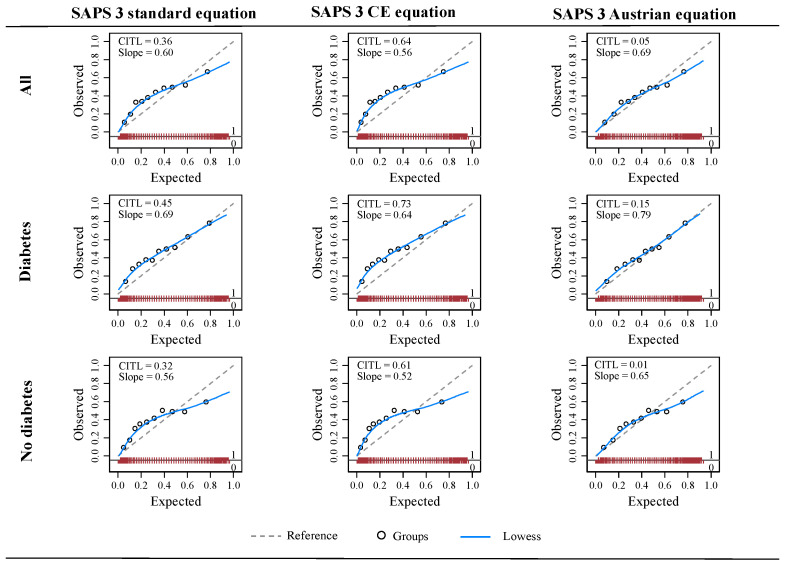
Calibration plots for SAPS 3 standard, SAPS 3 Central Europe, and SAPS 3 Austrian equations in all, diabetes, and non-diabetes patients with COVID-19. CE, Central European; CITL, calibration in-the-large; SAPS 3, simplified acute physiology score 3; Slope, calibration slope.

The SAPS 3 standard and Central European equations were miscalibrated in all three patient cohorts. Both equations underpredicted the mortality in low- and medium-risk groups and overpredicted the mortality in high-risk groups of all and non-diabetes patients. In patients with diabetes, these equations under-predicted the mortality in low- and medium-risk strata. In comparison, the Austrian recalibrated equation overpredicted the mortality in high-risk groups in the entire cohort and non-diabetes patients but had good calibration in low- and medium-risk strata. It showed reasonable calibration across all risk strata of diabetes patients as indicated by the calibration curve and H-L test (*p* = 0.339) (Table 3, Figure 3).

## 4. Discussion

This countrywide retrospective cohort analysis assessed and compared the performance of SAPS 3 for predicting the mortality in COVID-19 patients with and without diabetes using the standard and customized equations for Central Europe and Austrian COVID-19 patients. The standard and Central European equations significantly underestimated the in-hospital mortality in all three patient populations, while the Austrian equation accurately predicted in-hospital mortality in all three patient populations. The discrimination of all SAPS 3 equations was unsatisfactory in all patient cohorts and was insignificantly higher in patients with diabetes compared to those without diabetes. Likewise, the forecasting accuracy of all SAPS 3 equations was low in all cohorts. The calibration was poor for SAPS 3 standard and Central Europe equations in all three patient cohorts, and it was the worst in non-diabetes patients. The Austrian equation showed superior calibration to other SAPS 3 equations in all three populations; however, its calibration was satisfactory in diabetes patients only.

Our analysis revealed that both uncalibrated and calibrated versions of SAPS 3 equations demonstrated unsatisfactory discriminatory performance (AUC = 0.69) and accuracy (Brier score > 0.20) in patients with COVID-19. Although the performance of various prognostication scores has been evaluated in COVID-19 patients, surprisingly, only a few studies have validated the SAPS 3 in this patient population. Compared to our study, a recent research letter reported the discrimination of SAPS 3 (AUC = 0.75) in 1464 patients admitted to ICUs in Austria. However, it remarkably underestimated the in-hospital mortality (SMR = 1.20) especially in low-risk groups, thereby questioning its clinical applicability [6]. Another research letter showed that the discrimination of the SAPS 3 regional equation was good (AUC = 0.83) with a well-concorded SMR (0.95) in Brazilian COVID-19 patients [5]. We speculate that the SAPS 3 tool has yielded different discrimination in Brazilian and Austrian patients due to differences in healthcare infrastructure and other healthcare-related factors, treatment regimens, the severity of disease, and the distribution of these risk factors in the population under study [8]. In addition, the underlying risk factors and the magnitude of their coefficients that comprise the tool are central to the discriminatory performance of a tool [9]. Furthermore, the SAPS 3 simplifies significant factors such as old age [10] and physiological disturbances into categories, which may provide inappropriate coefficients of associations for predicting in-hospital mortality in COVID-19 patients [4]. This particular issue for some SAPS 3 risk factors was highlighted by a multicenter European study [11]. Moreover, COVID-19 is more prevalent in people with multimorbidity and affects multiple body systems, and several inflammatory, coagulation, and cardiac markers have been shown to predict its severity and adverse outcomes [12]. However, the SAPS 3 score does not incorporate all these factors and markers into its equation, which might have resulted in underpredicting the mortality and henceforth its poor predictive performance in COVID-19 patients [13,14].

While validating the performance of risk tools in a specific population, satisfactory discrimination alone does not guarantee that the very tool performs well in different risk strata of patients. For this reason, achieving an optimal calibration is equally important for accurately classifying patients into risk strata and henceforth making accurate clinical decisions. Considering that COVID-19 is a debilitating infection with a high mortality rate, the accurate identification of high-risk COVID-19 patients could be vital for their clinical management and prognosis. However, in our study, the SAPS 3 standard and Central European equations were extremely miscalibrated particularly in low- and medium-risk strata of patients. These findings are not surprising, as previous validation studies also found inadequate calibration for SAPS 3 in the Austrian and Brazilian COVID-19 patients. However, in the Brazilian COVID-19 patients, the miscalibration was more obvious in high-risk groups, while similar to our findings, it was more apparent in low-risk groups in the Austrian COVID-19 patients [5,6]. The issue of miscalibration for SAPS 3 standard equations has been well documented in various patient populations [11,15,16], which indicates that this tool does not perform well in specific populations due to various patient characteristics, healthcare-related factors, variability in the coefficient of association between some risk factors and mortality, and the level of predicted outcome in the population [11]. Consequently, poor calibration of SAPS 3 compromises its clinical utility in COVID-19 patients, a fact that clinicians should be aware of.

Given the above-mentioned reasons, recalibration of the SAPS 3 has been recommended prior to applying to any patient population [5,6]. Therefore, we also adopted the recently published SAPS 3 equation for COVID-19 patients to evaluate its predictive performance in our cohort of COVID-19 patients [6]. As expected, this equation was superior to standard and Central European equations for predicting the mortality as indicated by the SMR close to 1. However, interestingly, this equation overpredicted the mortality in high-risk groups in the entire cohort and non-diabetes patients but exhibited satisfactory calibration in patients with diabetes. As mentioned earlier, the uncalibrated equations showed a similar pattern of miscalibration in the Austrian COVID-19 patients in our study and the previous study [6]. Hence, it is possible that recalibrating the equation specifically for low-risk groups might have induced the miscalibration in high-risk groups as shown in our study. The selective adequate calibration of this SAPS 3 equation in diabetes patients is a conundrum when, in fact, diabetes is not included as a risk factor in this tool. We can only conjecture that people with diabetes are more likely to have severe COVID-19 disease, a higher burden of multimorbidity and risk factors, and pronounced physiological disturbances than their counterparts [13,15]. Perhaps that is why even uncalibrated equations showed better calibration in these patients. Nevertheless, our findings suggest that even the recalibrated equation of SAPS 3 have performed inadequately in COVID-19 patients, and therefore, this tool ought to be used with caution in this population. 

As stated above, people with diabetes may experience severe COVID-19 infection, its complications, and mortality due to compromised immune and inflammatory response, advanced age, multimorbidity, and metabolic derangements [13,17,18]. Hence, we expected that the SAPS 3 will exhibit superior discriminatory performance in patients with diabetes in comparison with non-diabetes. On the contrary, the discrimination was only ~2% (*p* = 0.193) higher in patients with diabetes than without diabetes. One probable reason for the similar discrimination might be related to the inherent risk factors that are considered in the calculation of SAPS 3 score. To elaborate, the SAPS 3 is not designed for any specific disease. Rather, it is based on comprehensive patient characteristics, previous health status and therapeutic interventions, surgical status, and physiological markers, which are not specific to diabetes and hence could be altered in ICU patients with any pathophysiological condition [4]. These afore-mentioned reasons further support our findings that SAPS 3 may not be an appropriate prognostic tool for many clinical conditions including COVID-19.

This study has several limitations. First, diabetes was not classified into type 1 and type 2 diabetes because of the issue of miscoding in ICD-10 codes. Second, in-hospital mortality was defined as death occurring from any underlying causes. This could have included non-COVID-19-related deaths. Nevertheless, as this database captures data for COVID-19 patients only, the probability of including other causes of death is minimal. Third, the predictive performance of the SAPS 3 is significantly influenced by characteristics of patients, distribution of risk factors comprising the SAPS 3 score, and the healthcare system under study. Therefore, the findings of our study may not be transferable to other COVID-19 cohorts.

## 5. Conclusions

To conclude, SAPS 3 showed low discrimination and accuracy in Austrian COVID-19 patients, which was insignificant between diabetes and non-diabetes patients. Both uncalibrated and European calibrated equations of SAPS 3 were extremely miscalibrated especially in non-diabetes patients. We therefore recommend investigating specific determinants of SAPS 3 discrimination and calibration in COVID-19 patients. Moreover, even though the Austrian equation calibrated for COVID-19 patients demonstrated a better calibration especially in patients with diabetes, its low discrimination and forecasting power suggests that even calibrated SAPS 3 versions should be administered with caution in COVID-19 patients and revalidated locally. In addition, it would be prudent to re-evaluate its predictive performance periodically and update it as required to incorporate the impact of changes in the SARS-CoV-2 virus characteristics and treatment regimens. Furthermore, as both standard and recalibrated equations of SAPS 3 demonstrated better predictive performance in COVID-19 patients with diabetes compared to non-diabetes patients, we recommend further studies investigating this phenomenon.

## Figures and Tables

**Table 1:** Characteristics of COVID-19 patients admitted to intensive care units, overall, and by diabetes status.

Variable	All	Diabetes		*p*-Value
		Yes	No	
All, *n* (%)	5850	1667 (28.50)	4183 (71.50)	--
Sex, *n* (%)				
Female	1985 (33.93)	541 (32.45)	1444 (34.52)	0.132
Male	3865 (66.07)	1126 (67.55)	2739 (65.48)	
Age, years, *n* (%)				
<40	215 (3.68)	22 (1.32)	193 (4.61)	<0.001
40–59	1258 (21.50)	330 (19.80)	928 (22.19)	
60–69	1471 (25.15)	457 (27.41)	1014 (24.24)	
70–74	906 (15.49)	312 (18.72)	594 (14.20)	
75–79	895 (15.30)	259 (15.54)	636 (15.20)	
≥80	1105 (18.89)	287 (17.22)	818 (19.56)	
Stay in hospital before ICU admission, days, *n* (%)				
<14	4437 (75.85)	1254 (75.22)	3183 (76.09)	0.781
14–27	912 (15.59)	267 (16.02)	645 (15.42)	
≥28	501 (8.56)	146 (8.76)	355 (8.49)	
Intra-hospital location before ICU admission, *n* (%)				
Operative room	334 (5.71)	70 (4.20)	264 (6.31)	0.017
Emergency room	920 (15.73)	265 (15.90)	655 (15.66)	
Other ICU	1160 (19.83)	344 (20.64)	816 (19.51)	
Hospital wards	3436 (58.74)	988 (59.27)	2448 (58.52)	
Comorbidities				
Cancer therapy, *n* (%)	480 (8.21)	154 (9.24)	326 (7.79)	0.069
Congestive heart failure, NYHA IV, *n* (%)	131 (2.24)	49 (2.94)	82 (1.96)	0.022
Hematological cancer, *n* (%)	148 (2.53)	29 (1.74)	119 (2.84)	0.015
Cirrhosis, *n* (%)	86 (1.47)	25 (1.50)	61 (1.46)	0.905
AIDS, *n* (%)	3 (0.05)	3 (0.18)	0 (0.00)	0.023
Cancer with metastasis, *n* (%)	114 (1.95)	16 (0.96)	98 (2.34)	0.001
Vasoactive drugs before ICU admission, *n* (%)	916 (15.66)	246 (14.76)	670 (16.02)	0.231
Reasons for ICU admission				
Cardiovascular, *n* (%)				
Arrhythmia	112 (1.91)	20 (1.20)	92 (2.20)	0.043
All others	5576 (95.32)	1601 (96.04)	3975 (95.03)	
Hypovolemic shock	32 (0.55)	6 (0.36)	26 (0.62)	
Septic, anaphylactic, undefined, and mixed shock	130 (2.22)	40 (2.40)	90 (2.15)	
Hepatic, *n* (%)				
All other	5831 (99.68)	1660 (99.58)	4171 (99.71)	0.420
Liver failure	19 (0.32)	7 (0.42)	12 (0.29)	
Digestive, *n* (%)				
All others	5783 (98.85)	1653 (99.16)	4130 (98.73)	0.260
Acute abdomen, other	54 (0.92)	10 (0.60)	44 (1.05)	
Severe pancreatitis	13 (0.22)	4 (0.24)	9 (0.22)	
Neurologic, *n* (%)				
Seizures	19 (0.32)	1 (0.06)	18 (0.43)	0.007
All others	5482 (93.71)	1562 (93.70)	3920 (93.71)	
Coma, stupor, obtund patient, agitation, vigilance disturbances, confusion, delirium	248 (5.24)	85 (5.10)	163 (3.90)	
Focal neurological deficit	71 (1.21)	12 (0.72)	59 (1.41)	
Intracranial mass effect	30 (0.51)	7 (0.42)	23 (0.55)	
Surgical status at ICU admission, *n* (%)				
No surgery	5251 (89.76)	1552 (93.10)	3699 (88.43)	<0.001
Scheduled surgery	294 (5.03)	64 (3.84)	230 (5.50)	
Emergency surgery	305 (5.21)	51 (3.06)	254 (6.07)	
Anatomical site of surgery, *n* (%)				
Transplant surgery	1 (0.02)	0 (0.00)	1 (0.02)	0.169
Trauma	51 (0.87)	11 (0.66)	40 (0.96)	
Cardiac surgery	23 (0.39)	6 (0.36)	17 (0.41)	
All others	5746 (98.22)	1647 (98.80)	4099 (97.99)	
Neurosurgery	29 (0.50)	3 (0.18)	26 (0.62)	
GCS score, median (IQR)	15 (1)	15 (1)	15 (1)	0.016
Mean ± SD	13.40 ± 3.51	13.34 ± 3.50	13.43 ± 3.51	
Total bilirubin, mg/dL, median (IQR)	0.60 (0.50)	0.50 (0.40)	0.60 (0.50)	<0.001
Body temperature, °C, mean ± SD	37.27 ± 1.30	37.31 ± 1.29	37.26 ± 1.21	0.192
Creatinine, mg/dL, median (IQR)	1.00 (0.60)	1.10 (0.90)	1.00 (0.60)	<0.001
Heart rate, bpm, mean ± SD	90 ± 30	93 ± 30	90 ± 29	<0.001
Leukocytes, G/L, median (IQR)	9.40 (6.40)	9.40 (6.20)	9.40 (6.50)	0.775
Hydrogen ion, pH, median (IQR)	7.42 (0.12)	7.41 (0.13)	7.42 (0.11)	<0.001
Platelets, G/L, median (IQR)	218.00 (122.00)	224.00 (123.50)	215.50 (123.00)	0.043
Systolic blood pressure, mmHg, mean ± SD	116.12 ± 31.27	116.68 ± 32.35	115.89 ± 30.81	0.417
PaO_2_, mmHg, median (IQR)	69 (27)	68 (26)	69 (28)	0.052
FiO_2_, %, median (IQR)	60 (40)	65 (30)	60 (40)	<0.001
SAPS 3 score, mean ± SD	57.39 ± 13.18	58.78 ± 12.92	56.84 ± 13.23	<0.001

GCS, Glasgow Coma Scale; ICU, intensive care unit; FiO_2_, fraction of inspired oxygen; PaO_2_, partial pressure of oxygen in arterial blood; SAPS 3, simplified acute physiology score 3.

**Table 2:** Comparison of observed in-hospital mortality in COVID-19 patients admitted to intensive care unit with diabetes and SAPS 3 variables.

Characteristic	In-Hospital Mortality		*p*-Value
	Yes	No	
All, *n* (%)	2276 (38.91)	3574 (61.09)	--
Diabetes, *n* (%)			
No	1560 (37.29)	2623 (62.71)	<0.001
Yes	716 (42.95)	951 (57.05)	
Sex, *n* (%)			
Female	742 (37.38)	1243 (62.62)	0.086
Male	1534 (39.69)	2331 (60.31)	
Age, years, *n* (%)			
<40	22 (10.23)	193 (89.77)	<0.001
40–59	219 (17.41)	1039 (82.59)	
60–69	496 (33.72)	975 (66.28)	
70–74	401 (44.26)	505 (55.74)	
75–79	467 (52.18)	428 (47.82)	
≥80	671 (60.72)	434 (39.28)	
Stay in hospital before ICU admission, days, *n* (%)			
<14	2009 (45.28)	2428 (54.72)	<0.001
14–27	197 (21.60)	715 (78.40)	
≥28	70 (13.97)	431 (86.03)	
Intra-hospital location before ICU admission, *n* (%)			
Operative room	64 (19.16)	270 (80.84)	<0.001
Emergency room	326 (35.43)	594 (64.57)	
Other ICU	479 (41.29)	681 (58.71)	
Hospital wards	1407 (40.95)	2029 (59.05)	
Comorbidities			
Cancer therapy, *n* (%)	247 (51.46)	233 (48.54)	<0.001
Congestive heart failure, NYHA IV, *n* (%)	84 (64.12)	47 (35.88)	<0.001
Hematological cancer, *n* (%)	72 (48.65)	76 (51.35)	0.003
Cirrhosis, *n* (%)	52 (60.47)	34 (39.53)	0.001
AIDS, *n* (%)	0 (0.00)	3 (100.00)	0.167
Cancer with metastasis, *n* (%)	60 (52.63)	54 (47.37)	0.002
Vasoactive drugs before ICU admission, *n* (%)	429 (46.83)	487 (53.17)	<0.001
Reasons for ICU admission			
Cardiovascular, *n* (%)			
Arrhythmia	35 (31.25)	77 (78.75)	<0.001
All others	2155 (38.35)	3421 (61.35)	
Hypovolemic shock	15 (46.88)	17 (53.12)	
Septic, anaphylactic, undefined, and mixed shock	71 (54.62)	59 (45.38)	
Hepatic, *n* (%)			
All other	1970 (33.78)	3861 (66.22)	<0.001
Liver failure	15 (78.95)	4 (21.05)	
Digestive, *n* (%)			
All others	2262 (39.11)	3521 (60.89)	0.003
Acute abdomen, other	9 (16.67)	45 (83.33)	
Severe pancreatitis	5 (38.46)	8 (61.54)	
Neurologic, *n* (%)			0.045
Seizures	6 (31.58)	13 (68.62)	
All others	2115 (38.58)	3367 (61.42)	
Coma, stupor, obtunded patient, agitation, vigilance disturbances, confusion, delirium	119 (47.98)	129 (52.02)	
Focal neurological deficit	25 (35.21)	46 (64.79)	
Intracranial mass effect	11 (36.67)	19 (63.33)	
Surgical status at ICU admission, *n* (%)			<0.001
No surgery	2155 (41.04)	3096 (58.96)	
Scheduled surgery	47 (15.99)	247 (84.01)	
Emergency surgery	74 (24.26)	231 (75.74)	
Anatomical site of surgery, *n* (%)			
Transplant surgery	1 (100.00)	0 (0.00)	<0.001
Trauma	14 (27.45)	37 (72.55)	
Cardiac surgery	0 (0.00)	23 (100.00)	
All others	2255 (39.24)	3491 (60.76)	
Neurosurgery	6 (20.69)	23 (79.31)	
GCS score, median (IQR)	15 (2)	15 (0)	<0.001
Mean ± SD	12.72 ± 3.99	13.83 ± 3.08	
Total bilirubin, mg/dL, median (IQR)	0.60 (0.50)	0.60 (0.40)	0.001
Body temperature, °C, mean ± SD	37.26 ± 1.29	37.29 ± 1.20	0.471
Creatinine, mg/dL, median (IQR)	1.19 (0.80)	0.90 (0.50)	<0.001
Heart rate, bpm, mean ± SD	97 ± 26	93 ± 23	<0.001
Leukocytes, G/L, median (IQR)	9.87 (7.10)	9.00 (5.80)	<0.001
Hydrogen ion, pH, median (IQR)	7.40 (0.14)	7.43 (0.09)	<0.001
Platelets, G/L, median (IQR)	202.00 (117.00)	229.00 (123.00)	<0.001
Systolic blood pressure, mmHg, mean ± SD	112.57 ± 31.93	118.43 ± 30.62	<0.001
PaO_2_, mmHg, median (IQR)	66 (24)	70 (29)	<0.001
FiO_2_, %, median (IQR)	70 (40)	55 (40)	<0.001
SAPS 3 score			
All patients, mean ± SD	62.57 ± 12.86	54.10 ± 12.29	<0.001
Diabetes, mean ± SD	63.96 ± 13.15	54.87 ± 11.28	<0.001
No diabetes, mean ± SD	61.92 ± 12.68	53.82 ± 12.62	<0.001

GCS, Glasgow Coma Scale; ICU, intensive care unit; FiO_2_, fraction of inspired oxygen; PaO_2_, partial pressure of oxygen in arterial blood; SAPS 3, simplified acute physiology score 3.

**Table 3:** Performance of SAPS 3 standard, Central Europe, and Austrian equations in predicting in-hospital mortality in all, diabetes, and non-diabetes patients.

SAPS 3 Equations	Mortality		Discrimination	Calibration	
	Predicted Mortality Mean ± SD	SMR (95%CI)	AUROC (95%CI)	H-L X^2^, *p*-Value	Brier Score
Standard equation					
All	32.47 ± 21.69	1.20 (1.16–1.24)	68.67 (67.31–70.02)	100.03, <0.001	0.22
Diabetes	34.56 ± 21.62	1.24 (1.18–1.31)	70.03 (67.53–72.53)	12.21, 0.142	0.22
No diabetes	31.63 ± 21.66	1.18 (1.13–1.22)	68.05 (66.44–69.67)	101.64, <0.001	0.22
Central Europe equation					
All	28.05 ± 21.43	1.39 (1.34–1.43)	68.67 (67.31–70.02)	120.95, <0.001	0.23
Diabetes	30.02 ± 21.56	1.43 (1.35–1.51)	70.03 (67.53–72.53)	15.08, 0.058	0.23
No diabetes	27.28 ± 21.33	1.37 (1.31–1.42)	68.05 (66.44–69.67)	119.99, <0.001	0.23
Austrian equation					
All	37.86 ± 20.56	1.03 (0.99–1.06)	68.67 (67.31–70.02)	65.10, <0.001	0.22
Diabetes	40.03 ± 20.16	1.07 (1.02–1.13)	70.03 (67.53–72.53)	9.04, 0.339	0.22
No diabetes	37.00 ± 20.66	1.01 (0.98–1.05)	68.05 (66.44–69.67)	69.55, <0.001	0.22

AUROC, area under the receiver operating characteristic curve; CI, confidence interval; H-L X^2^, Hosmer–Lemeshow chi-square test; SAPS 3, simplified acute physiology score 3; SMR, standardized mortality ratio.

## Data Availability

The dataset used in this study is a property of the Austrian National Public Health Institute (Gesundheit Österreich GmbH). Further information regarding data access is available at: https://datenplattform-covid.goeg.at/english, accessed on 3 May 2021.
